# Editorial: Women in pediatric obesity 2022

**DOI:** 10.3389/fped.2023.1273635

**Published:** 2023-08-23

**Authors:** Megan L. Gow, Annalisa Terranegra, Melania Manco

**Affiliations:** ^1^ Children's Hospital Westmead Clinical School, The University of Sydney Children's Hospital Westmead Clinical School, Sydney, NSW, Australia; ^2^ Discipline of Paediatrics, School of Clinical Medicine, UNSW Medicine and Health, University of New South Wales, Sydney, NSW, Australia; ^3^ Translational Medicine Department, Sidra Medicine, Doha, Qatar; ^4^ Scientific Directorate, Bambino Gesù Hospital and Research Institute, Rome, Italy

**Keywords:** obesity, pediatrics-children, editorial, women in STEM, pediatric obesity

**Editorial on the Research Topic** Women in pediatric obesity 2022

## 1. Introduction

At present, less than 30% of researchers worldwide are women. Long-standing biases and gender stereotypes are discouraging girls and women away from science-related fields, including science, technology, engineering, and mathematics (STEM) research. Science and gender equality are, however, essential to ensuring sustainable development as highlighted by UNESCO. In order to change traditional mindsets, gender equality must be promoted, stereotypes defeated, and girls and women encouraged to pursue STEM and research careers.

Our Research Topic promotes the work of women scientists, across all fields of pediatric obesity. To be considered for this collection, the first or last author had to be a researcher who identifies as a woman.

## 2. Outline of contributions

In total, our Research Topic accepted six manuscripts from contributors in the United States (*n* = 3), China (*n* = 1), Denmark (*n* = 1), and Ireland (*n* = 1). Accepted manuscripts covered a range of topics including gestational (*n* = 1), parental (*n* = 1), and behavioral (*n* = 1) determinants of obesity, assessing the management of pediatric obesity in inpatient (*n* = 1) and primary care settings (*n* = 1), and a randomized controlled trial (RCT) of a family-based pediatric obesity prevention program (*n* = 1).

The most viewed article in the collection to date is the RCT published by Teran-Garcia and colleagues (1,212 views). This multi-center community program for Mexican and Puerto Rican families evaluated the effect of six 2-hour family-based workshops to reduce the BMI *z*-score in 239 children aged 6–18 years, compared with 187 children of families who received printed generic nutrition and wellness information. At baseline, there were no differences between groups, and post-intervention BMI *z*-scores decreased in the intervention group (−0.03, 95% CI: −0.066, −0.003, *P* = 0.032) but not in the control group. Six months post-intervention, there were no differences between groups. These findings suggest that culturally tailored programs can provide families with the knowledge to produce short-term changes. However, additional efforts are required to sustain changes longer-term.

Three articles in the collection focused on various determinants of pediatric obesity. A study from Denmark used data from the APPROACH study (an optimized programming of healthy children) to investigate whether limiting gestational weight gain to 9 kg (based on Institute of Medicine guidelines) in women who have pre-pregnancy overweight or obesity (BMI 28–45 kg/m^2^) impacted on infant birth weight and BMI *z*-score. Infants born by mothers whose GWG was ≤9 kg weighed less (122 g, 95% CI: 6–249, *P* = 0.040), had similar BMI *z*-scores (0.2 SD, 95% CI: −0.06 to 0.55, *P* = 0.120), and lower incidence of emergency cesarean deliveries (11.5% vs. 23.1%, *P* = 0.044) compared to infants born by mothers whose GWG was >9 kg. The authors suggest that limiting weight gain during pregnancy in women who have pre-pregnancy overweight or obesity may have a role in preventing future obesity in the infant.

In a prospective cohort study including 245 children (80 non-Hispanic white, 23 non-Hispanic black, 104 Hispanic/Latinx) living in Washington, DC; Baltimore, MD; and Lake County, IL in the Unites States, maternal and paternal factors were assessed for their association with changes in children's BMI percentile from 12 to 24 months of age. Changes in BMI percentile varied by race/ethnicity. Among white children, greater parental education was associated with greater decreases in BMI percentile. Among Hispanic/Latinx children, older maternal age, younger paternal age, and child female sex were associated with gains in BMI percentile. While the sample size of this study was limited, especially for non-Hispanic black participants, the findings highlight the need to consider potential racial differences in the development of obesity in children.

The third study looking at determinants of obesity was that by Dai et al. from China, which examined whether eating meats/fish or vegetables first as part of a meal impacted on the risk of obesity in 2,049 preschoolers. Overall, there was no difference in the BMI *z*-score of children eating meats/fish first vs. children eating vegetables first during a meal. Notably, children with parents who were affected by obesity were more likely to eat vegetables first. Among children of mothers with obesity, the child BMI *z*-score was significantly higher in the meats/fish first group than the vegetable first group (2.891 vs. 0.845, *P* = 0.007). In children whose mothers were affected by obesity, those that ate meats/fish first had a 12.21 times higher risk of being overweight compared with those that ate vegetables first (95% CI: 1.22–121.74, *P* = 0.033). The authors suggested that eating vegetables first during a meal may be recommended for children whose mothers are affected by obesity.

The final two studies in the collection examined aspects of medical management of pediatric obesity; one reviewed the existing literature on inpatient weight loss treatments and presented three examples of acute weight loss in hospitalized pediatric patients, and the other mapped the perceived barriers and facilitators related to pediatric obesity management across healthcare settings, professional disciplines, and regions in the Republic of Ireland. The review identified limited studies addressing inpatient pediatric weight loss treatments but found that existing evidence suggests that weight management can and should be considered in an inpatient setting, either as the primary outcome or as an adjunct to other medical treatment. The findings indicate that further investigation is required to design and implement inpatient weight management protocols for youth with obesity while hospitalized.

The Irish study highlighted that there are gaps in the understanding of guidelines and dissimilarity in assessment, counselling, and treatment practices by practitioners in the Republic of Ireland for childhood obesity management. To improve professionals' self-efficacy, the authors suggest that healthcare professionals must access clinical training and education so that evidence-based treatment can be delivered to maximize health outcomes for children with obesity.

## 3. Concluding remarks

Our Research Topic presents a range of manuscripts led by women researchers working in pediatric obesity and represents a diverse collection of research across the spectrum of pediatric obesity research, including predictors of pediatric obesity, prevention interventions, and assessment of current management. Women in STEM have further opportunities to highlight their research in Frontiers in Pediatrics, with submissions currently open for the Research Topics “Women in Pediatric Critical Care: 2023”, manuscript submission deadline 6 October 2023, and “Women in Obstetric and Pediatric Pharmacology: 2023”, manuscript submission deadline 23 September 2023.

